# Heavy metal contamination in duck eggs from a mercury mining area, southwestern China

**DOI:** 10.3389/fpubh.2024.1352043

**Published:** 2024-02-28

**Authors:** Xiaoling Guo, Zhuhong Wang, Xue Li, Jing Liao, Xue Zhang, Yulin Ran, Qixin Wu, Ting Zhang, Zhongwei Wang

**Affiliations:** ^1^School of Public Health, The Key Laboratory of Environmental Pollution Monitoring and Disease Control, Ministry of Education, Guizhou Medical University, Guiyang, China; ^2^Xishui County Center for Disease Control and Prevention, Xishui, Guizhou, China; ^3^Key Laboratory of Karst Georesources and Environment, Ministry of Education, Guizhou University, Guiyang, China; ^4^Guangdong Ecological and Environmental Monitoring Center, Guangzhou, China

**Keywords:** heavy metal, health risk, mercury mine area, free-range duck eggs, caged duck eggs

## Abstract

**Objective:**

Mercury (Hg) contamination in the environment around mercury mines is often accompanied by heavy metal contamination.

**Methods:**

Here, we determined concentrations of chromium (Cr), zinc (Zn), strontium (Sr), barium (Ba), and lead (Pb) in duck eggs from a Hg mining area in Southwest China to assess the contamination and health risk.

**Results:**

Duck eggs obtained from the mining area exhibit higher concentrations of Cr, Zn, Sr, Ba, and Pb compared to those from the background area, with egg yolks containing higher metal levels than egg whites. Specifically, the mean Cr, Zn, Sr, Ba, and Pb concentrations of duck eggs from the Hg mining area are 0.38, 63.06, 4.86, 10.08, and 0.05 μg/g, respectively, while those from the background area are only 0.21, 24.65, 1.43, 1.05, and 0.01 μg/g. Based on the single-factor contamination index and health risk assessment, heavy metal contamination in duck eggs poses an ecological risk and health risk.

**Conclusion:**

This study provides important insight into heavy metal contamination in duck eggs from Hg mining areas.

## 1 Introduction

Heavy metal pollution can pose serious harm to wildlife and human beings. Mercury (Hg), a globally transported heavy metal with strong bioaccumulation, is found in excessive levels in the atmosphere, water, soil, vegetables, and rice in Hg mining areas (1). Hg pollution is often accompanied by other heavy metal contaminations in Hg mining areas (2). Soil, water and crops in Hg mining areas are contaminated with heavy metals to varying degrees (3–5). For instance, cadmium (Cd) and arsenic (As) levels in the soil exceed the standard limits in Wuchuan mining area, SW China (2). A previous study revealed high heavy metal (including Hg, As, Cd and Se) levels in eight types of vegetables in mining regions (6). Excessive ingestion of heavy metals can be toxic (7, 8). Cr has mutagenic, teratogenic, and carcinogenic properties (9). Pb and Hg are known to have neurotoxic effects, particularly harmful to the neurological development of children (10). Excessive amounts of Zn, Sr, and Ba have been found to induce genotoxic effects in cells (11). Given the potential harm heavy metals may pose to humans, heavy metal pollution in mining areas cannot be overlooked (6).

Consumption of poultry products could be an important exposure of heavy metals to humans. Poultry normally uptake heavy metals from various sources (e.g., feed, water), among which feed is the main source, and the females can transfer heavy metals to their eggs (12). Normally, farmed poultry eat a fixed recipe of feed, while free-range poultry mainly ingest local crops (12). However, most studies on health risk assessments of heavy metals focus on commercial (13), selenium-enriched (14), and free-range chicken eggs (15), with limited research on duck eggs. It is worth noting that China is the largest producer and consumer of duck eggs in the world, with an output of ~4 million tons annually (16, 17). A meta-analysis indicates that duck eggs contain higher levels of potentially toxic elements compared to chicken eggs (18). Due to the high Hg level in poultry eggs from Wuchuan compared to other areas, and the total Hg concentration in duck eggs exceeds that of chicken eggs (19), we hypothesize that concentrations of other heavy metals could be high in local poultry eggs (e.g., duck eggs). However, heavy metal (such as Zn, Cr, Sr, Ba and Pb) levels in local duck eggs and their potential harm to consumers are unclear so far. Therefore, it is crucial to assess the potential health risks associated with the consumption of duck eggs contaminated with heavy metals.

Here, we conducted the research on heavy metal concentrations in duck eggs from Wuchuan (a Hg mining area), to understand the heavy metal levels in duck eggs and their potential health risks in Hg mining area. This study could provide insights into the current of heavy metal levels in Wuchuan duck eggs and to assess the potential human health risks from consuming such duck eggs from the Hg mining area.

## 2 Materials and methods

### 2.1 Study area

**Figure 1:**
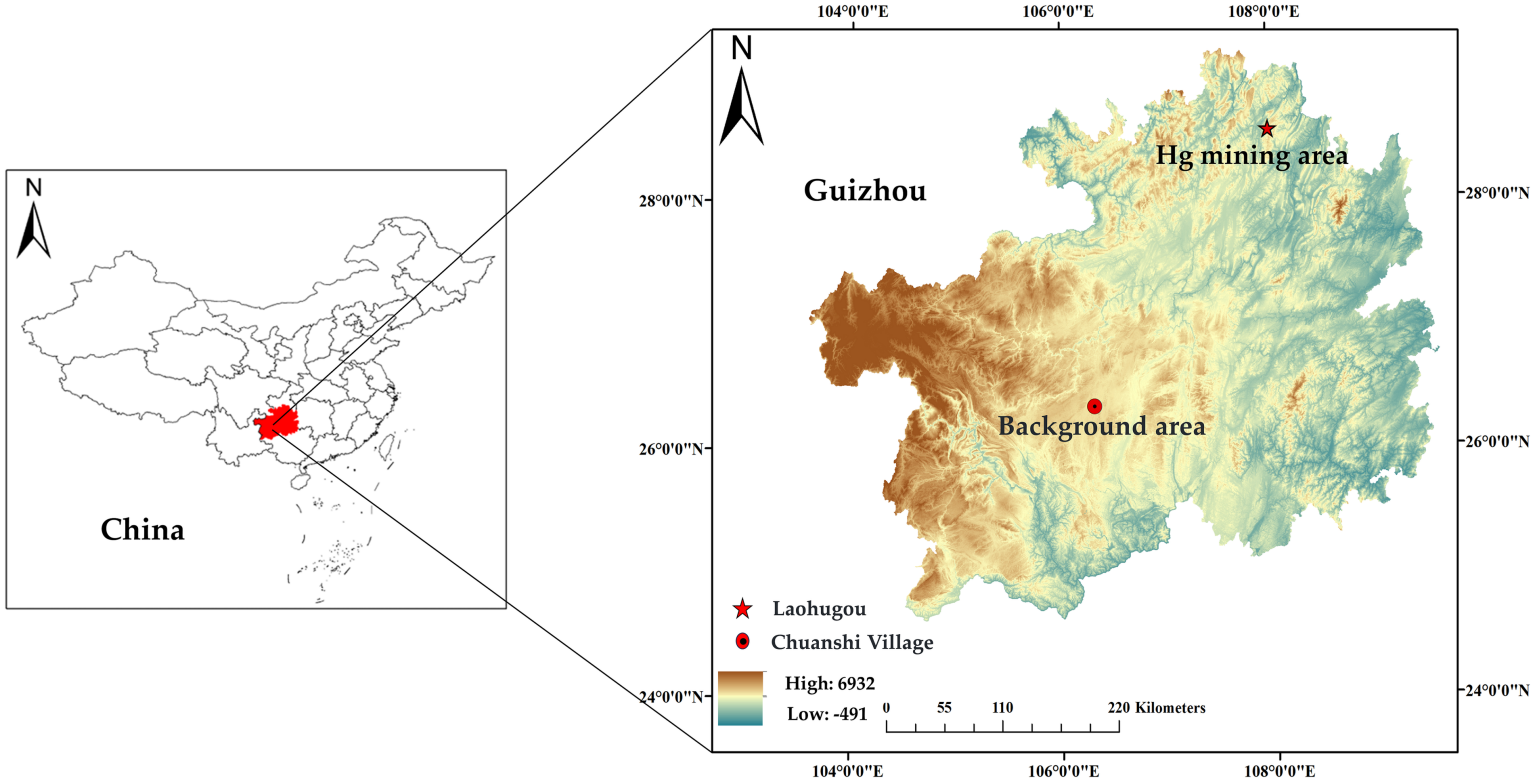
Distribution of duck egg sampling sites in the study area.

Wuchuan is located in Guizhou Province, southwestern China (Figure 1). Wuchuan Hg mine is one of the largest Hg mines in Guizhou Province, with a Hg production history of about 400 years (20, 21). Despite the cessation of mining for 20 years, local environmental Hg levels (in soil, water, and rice) are still evidently higher than the standard limitation (6). The concentrations of Hg are 1.3 ~ 360 mg/kg in local topsoil variation, 13 ~ 2,100 ng/L in water, and 6.0 ~ 113 in rice ng/g (22, 23). Different from Wuchuan, Hg and other heavy metals are within safe thresholds in Anshun (24, 25). In Anshun, Hg concentration in rice and other crops is about 0.01 mg/kg, significantly below the limit set by the “National Food Safety Standard for Contaminants in Foods” (24). Additionally, the level of heavy metal contamination in the soil is low (25).

We collected ten duck eggs each from Laohugou, Wuchuan County, Zunyi (28°60′ N, 108°01′ E) and Chuanshi Village, Yangchang Town, Anshun (26°35′ N, E 106°32′ E). As the ducks raised by surrounding residents in the mining area have similar feeding methods and feed, random sampling of purchased duck eggs was conducted. The eggs were from ducks raised by local households with free-range. Sampling locations, quantities, and times were shown in Table 1 (26). The collected duck eggs were brought to the laboratory within 24 h and stored at 4°C.

**Table 1:** Duck egg sampling location, time and number.

Sampling location	Sampling time	Number
Laohugou Mercury Mining Area, Wuchuan, Guizhou, China	July 18, 2022	10
Chuanshi Village, Yangchang Town, Anshun, Guizhou, China	July 25, 2022	10

### 2.2 Analysis method

#### 2.2.1 Materials and reagents

Process ultrapure grade HNO_3_, 30% H_2_O_2_ (superior purity), Milli-Q water (18.2 MΩ.cm, Millipore), ICP-MS (Thermos Scientific iCAP RQ).

#### 2.2.2 Pretreatment of samples

The duck eggs were washed with 18.2 MΩ water, followed by separation of egg yolk and egg white, and then freeze-dried and mixed. Each sample (0.500 g) was digested with 5 mL of nitric acid (process ultrapure) and 1 mL of hydrogen peroxide at 160°C for 8 h. After cooling to room temperature, the inner chamber of the digestion tank was removed and the inner lid was rinsed with a small amount of ultrapure water, and inner chamber placed on a hot plate at 90°C, 2% nitric acid fixed volume to 10 mL and stored at 4°C before measurement.

#### 2.2.3 Determination of metal concentrations and quality control

The heavy metals Cr, Zn, Sr, Ba, and Pb were determined by ICP-MS (Thermos Scientific iCAP RQ) at Guizhou University. Quality control was performed by standard reference materials (VAR-CAL-2 for trace elements; CLMS-1 for rare earth elements). The relative standard deviations of the metals were all below 10%, and the recoveries were 80% ~ 110%.

### 2.3 Evaluation of heavy metal pollution of duck eggs

According to the single factor pollution index, heavy metal contamination in duck eggs is evaluated as following:


(1) $$P_{i}=C_{i}/S_{i}$$

Where P_i_ is the single-factor pollution index, C_i_ is the concentration of metals in duck eggs (μg/g, DW), and S_i_ denotes the evaluation standard value of the five heavy metals in duck eggs (μg/g). Pb is the standard value according to the Chinese national standard for food safety (GB 2762–2022). Given the lack of standard limits for metals in duck eggs, the corresponding metal levels in the background area are used as the standard limit for other metals in this study. As shown in Supplementary Table S1 (27), are the grading standards.

### 2.4 Health risk assessment

The US Environmental Protection Agency (USEPA) health risk assessment model was used to assess non-carcinogenic and carcinogenic risks based on exposure parameters in the Chinese population (28). The noncarcinogenic risks for Cr, Zn, Sr, Ba, and Pb and the carcinogenic risks for Cr and Pb are calculated according to the International Agency for Research on Cancer (the International Agency for Cancer, 2020) carcinogen classification.

Heavy metal chronic daily intake can be calculated as following:


(2) $$EDI=\frac{C_{i}\times IR\times ED\times EF}{AT\times BW}$$

Where EDI is the estimated daily intake of heavy metals (mg/kg/day), $C_{i}$ is the concentration of metals in duck eggs (μg/g), IR is the dietary intake (kg/d), ED is the exposure time (a), EF is the exposure frequency (d/a), AT is the average exposure time (d), and BW is the body weight (kg). IR is 0.15 kg/d and 0.10 kg/d for adults and children, respectively; ED is 30 years and 10 years, respectively; EF are 365 d/a and 365 d/a, respectively; AT is 10950 d and 3,650 d, respectively; and BW are 70 kg and 16 kg, respectively (29–33).

The noncarcinogenic risk of consuming contaminated duck eggs is calculated and assessed by the health risk quotient (HQ) and the health risk index (HI, the sum of the HQ values of different metals, used to calculate the noncarcinogenic risk caused by multiple heavy metals). Heavy metal HQ and HI can be calculated as follows:

(3) $$HQ=\frac{EDI}{RfD}$$

(4) $$HI=\sum {HQ}_{i}$$

Where HQ is the one-factor noncarcinogenic risk index, EDI is the chronic daily intake of heavy metals (mg/kg/day), RfD is the reference consumption of heavy metals (0.003, 0.300, 0.600, 0.200, and 0.0035 mg/kg/day for Cr, Zn, Sr, Ba, and Pb, respectively), and HI is the total noncarcinogenic risk index for the five elements. HQ or HI > 1 indicates a potential non-carcinogenic risk, while HQ or HI < 1 indicates no potential risk (34–36).

The total carcinogenic risk (TCR) is the sum of the carcinogenic risk (CR) values of different metals. Heavy metal CR and TCR can be calculated as follows:


(5) CR=EDI×SF

(6) $$TCR=\sum {CR}_{i}$$

Where CR is the heavy metal carcinogenic risk index, EDI is the chronic daily intake of heavy metals (mg/kg/day), SF is the slope factor of carcinogenic heavy metals (0.005 and 0.0085 for Cr and Pb, respectively), and TCR is the total heavy metal carcinogenic risk index. When CR or TCR ≤ 1 × 10^−6^, the carcinogenic risk is considered negligible, while CR or TCR < 1 × 10^−4^ indicates a low risk and is considered acceptable, and CR or TCR ≥ 1 × 10^−4^ indicates a potential carcinogenic risk (37, 38).

### 2.5 Data analysis

ArcGIS is used to plot the distribution of sampling points, and SPSS 26.0 is used to analyze the data. In this study, we applied the Wilcoxon rank sum test uniformly to analyze the significant differences in metal concentrations and pollution index. Moreover, the mean, median, minimum, and maximum values of metal concentration and pollution index were shown.

## 3 Results

### 3.1 Metal pollutions

**Figure 2:**
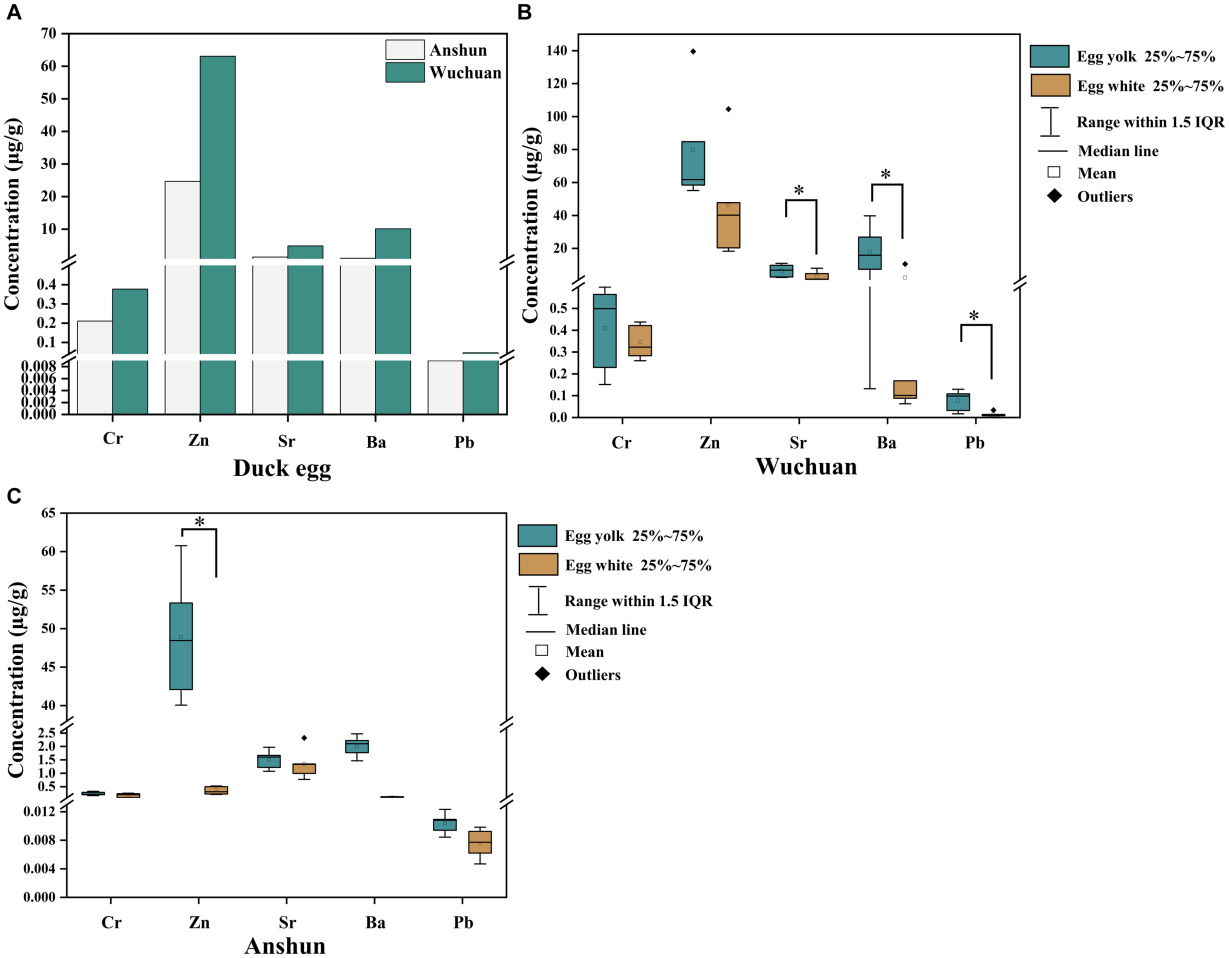
Heavy metal concentrations in duck egg yolk and egg white from the background area. **(A)** Cr, Zn, Sr, Ba, Pb concentrations in duck eggs from Wuchuan (Hg mining area) and Anshun (background area); **(B)** Cr, Zn, Sr, Ba, Pb concentrations in duck eggs from Wuchuan; **(C)** Cr, Zn, Sr, Ba, Pb concentrations in duck eggs from Anshun. “*” represents significantly different (*p* < 0.05).

The mean Cr, Zn, Sr, Ba, and Pb concentrations of duck eggs from the Hg mining area are 0.38, 63.06, 4.86, 10.08, and 0.05 μg/g, respectively, whereas those from the background area are 0.21, 24.65, 1.43, 1.05, and 0.01 μg/g, respectively. The concentrations of Cr, Zn, Sr, and Pb in duck eggs from the Hg mining area are significantly higher than those from the background area (*p* < 0.05; Figure 2A). Specifically, the mean concentrations of Cr, Zn, Sr, Ba, and Pb in egg yolk are 0.41, 79.90, 6.44, 17.98, and 0.08 μg/g, respectively, while the mean concentrations of Cr, Zn, Sr, Ba, and Pb in egg white are 0.35, 47.73, 7.62, 2.18, and 0.02 μg/g, respectively (Figure 2B; Table 2). In the Hg mining area, the mean concentrations of Sr, Ba, and Pb in the yolk are much higher than those in egg white (*p* < 0.05). In the background area, the mean concentrations of Cr, Zn, Sr, Ba, and Pb in egg yolk are 0.24, 48.94, 1.51, 2.01, and 0.01 μg/g, respectively. The mean concentrations of Cr, Zn, Sr, Ba, and Pb in egg white are 0.36, 3.75, 1.35, 0.10, and 0.01 μg/g, respectively. The mean concentration of Zn in egg yolk is also much higher than that in the duck egg whites from the background area (*p* < 0.05) (Figure 2C; Table 2). Overall, the concentrations of Cr, Zn, Sr, Ba, and Pb in duck eggs in the Hg mining area are higher than those in the background area, and the metal concentrations in yolks are higher than those in egg whites.

**Table 2:** The concentrations of Cr, Zn, Sr, Ba, and Pb in duck egg yolk and egg white at the Hg mining area and the background area.

Areas	Samples	Metals (μg/g)	Mean	Median	Min.	Max.
The Hg mining area	Egg yolk	Cr	0.41	0.50	0.15	0.60
		Zn	79.90	61.75	55.10	139.50
		Sr	6.44	6.73	2.34	10.85
		Ba	17.98	15.82	0.13	39.78
		Pb	0.08	0.10	0.02	0.13
	Egg white	Cr	0.35	0.32	0.26	0.44
		Zn	47.73	34.03	18.32	104.51
		Sr	2.87	1.27	1.08	7.87
		Ba	2.18	0.10	0.06	10.46
		Pb	0.01	0.01	0.01	0.03
The background area	Egg yolk	Cr	0.24	0.22	0.16	0.33
		Zn	48.94	48.45	40.07	60.77
		Sr	1.51	1.60	1.08	1.97
		Ba	2.01	2.10	1.46	2.47
		Pb	0.01	0.01	0.01	0.01
	Egg white	Cr	0.16	0.20	0.00	0.26
		Zn	0.35	0.31	0.21	0.53
		Sr	1.35	1.34	0.77	2.32
		Ba	0.10	0.10	0.05	0.13
		Pb	0.01	0.01	0.00	0.01

### 3.2 The duck eggs of single factor pollution index of heavy metals

**Figure 3:**
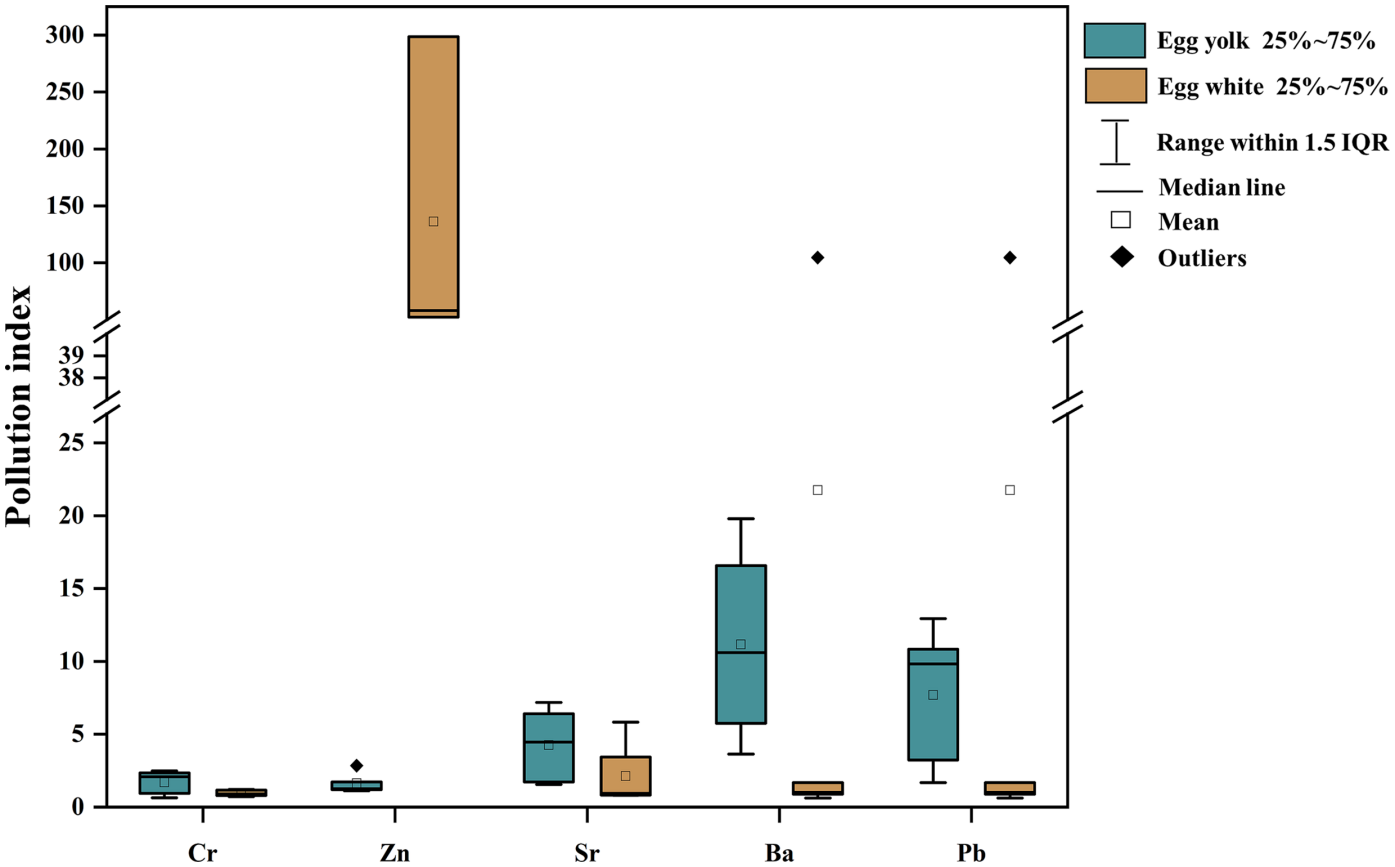
The pollution index of duck egg whites and yolks in the Wuchuan (Hg mining area).

The single factor pollution index is an indicator to evaluate a single factor in heavy metal pollution, which has already been used in evaluations of heavy metal pollution in various environmental media and materials, including water, soil, crops, etc. Given the lack of standards for the five metals in this study, we take Anshun as the background area and assess the heavy metal pollution in duck eggs in the mercury mining area based on the related calculation (Figure 3). The single factor pollution index in egg yolk declines in the following order: Pb (9.83) > Ba (7.87) > Sr (4.46) > Cr (2.07) > Zn (1.26), and in egg white is characterized as Zn (58.12) > Pb (1.31) > Ba (1.01) > Cr (0.90) > Sr (0.83) (Table 3). Except for the Cr and Sr. single factor contamination index in egg white which is less than 1, other heavy metals are all >1 in egg yolk and egg white, indicating that duck eggs are contaminated with heavy metals at different levels.

**Table 3:** The single factor pollution index of Cr, Zn, Sr, Ba, and Pb in duck egg yolk and egg white at the Hg mining area.

Samples	Metals (μg/g)	P_i_			
		Mean	Median	Min.	Max.
Egg yolk	Cr	1.70	2.07	0.63	2.49
	Zn	1.63	1.26	1.23	2.85
	Sr	4.27	4.46	1.55	7.18
	Ba	8.94	7.87	0.07	19.79
	Pb	7.70	9.83	1.68	12.94
Egg white	Cr	0.96	0.90	0.72	1.22
	Zn	81.82	58.12	52.34	298.61
	Sr	1.70	0.83	0.80	5.83
	Ba	21.76	1.01	0.63	104.57
	Pb	1.83	1.31	0.94	4.20

P_i_ is the single-factor pollution index, which is one of the indicators of ecological risk assessment.

### 3.3 Health risk assessment of heavy metals

#### 3.3.1 Noncarcinogenic risk assessment of heavy metals in duck eggs

Carcinogenic and noncarcinogenic health risk estimates are widely recognized as important parameters for human health risk assessment. Table 4 shows the heavy metal intake and noncarcinogenic risk in duck eggs from the Hg mining area. The mean daily intake (EDI) of Cr, Zn, Sr, and Pb in egg yolk for adults and the Cr, Zn, and Pb in egg white are less than the reference exposure dose (RfD), while EDI for Ba in egg yolk and Sr, Ba, and Pb in egg white are greater than RfD. Children EDI is less than the RfD for Cr and Pb in egg yolk and Cr, Zn, Sr, Ba, and Pb in egg white, while EDI for Zn, Sr, and Ba in egg yolk and Pb in egg white are greater than RfD. Furthermore, the total health risk index of egg yolk consumption is >1 for adults, while the index of both egg yolk and egg white are >1 for children, higher than that for adults.

**Table 4:** Noncarcinogenic risk assessment of heavy metals in duck eggs in the study area.

Metals		EDI		HQ		HI	
		Adult	Children	Adult	Children	Adult	Children
Cr	Egg yolk	0.8 × 10^−3^	2.6 × 10^−3^	0.29	0.85	1.13	3.28
	Egg white	0.7 × 10^−3^	2 × 10^−3^	0.25	0.72	0.49	1.43
Zn	Egg yolk	0.6 × 10^−1^	5.0 × 10^−1^	0.57	1.67	1.13	3.28
	Egg white	1.7 × 10^−1^	1.8 × 10^−1^	0.21	0.6	0.49	1.43
Sr	Egg yolk	0.1 × 10^−1^	4.0 × 10^−3^	0.02	0.07	1.13	3.28
	Egg white	4.9 × 10^−3^	1.4 × 10^−3^	0.01	0.02	0.49	1.43
Ba	Egg yolk	3.8 × 10^−2^	11.2 × 10^−3^	0.19	0.56	1.13	3.28
	Egg white	4.7 × 10^−3^	1.3 × 10^−3^	0.02	0.07	0.49	1.43
Pb	Egg yolk	1.6 × 10^−4^	0.4 × 10^−3^	0.05	0.14	1.13	3.28
	Egg white	3.14 × 10^−5^	9.17 × 10^−5^	0.01	0.03	0.49	1.43

#### 3.3.2 Carcinogenic risk assessment of heavy metals in duck eggs

Due to the lack of carcinogenic slope factors for Zn, Sr, and Ba, the carcinogenic risk assessment is only conducted for Cr and Pb. When the carcinogenic risk is greater than 1.00 × 10^−6^, it indicates a certain carcinogenic risk (39, 40). In this study, we observed that the carcinogenic risk of Cr and Pb in both yolk and white of duck eggs is >1.00 × 10^−6^. Cr has a greater carcinogenic risk than Pb (Table 5). It is noteworthy that the total carcinogenic risk of duck egg intake in children is greater than adults, and that of egg yolk is greater than egg white for both adults and children.

**Table 5:** Carcinogenic risk assessment of heavy metals in duck eggs from Hg mining areas.

Metals		CR		TCR	
		Adult	Children	Adult	Children
Cr	Egg yolk	4.38 × 10^−6^	1.28 × 10^−5^	5.78 × 10^−6^	16.86 × 10^−6^
	Egg white	3.70 × 10^−6^	1.08 × 10^−5^	3.96 × 10^−6^	11.56 × 10^−6^
Pb	Egg yolk	1.40 × 10^−6^	4.09 × 10^−6^	5.78 × 10^−6^	16.86 × 10^−6^
	Egg white	2.67 × 10^−7^	7.79 × 10^−7^	3.96 × 10^−6^	11.56 × 10^−6^

## 4 Discussion

### 4.1 Analysis of heavy metal pollution

In this study, preliminary results indicate that duck eggs in Hg mining areas are contaminated with Cr, Zn, Sr, Ba, and Pb. The higher metal concentration of duck eggs in the Hg mining area than in the background area is related to the high metal levels in the mining area environment. The tailings left after the cessation of mining impact the local soil and water, and the soil in the mining area is contaminated to varying degrees with Cd, Pb, Zn, Cr and As (7, 41, 42). Cr and As exceed the standard in the water of the mining area (8, 43). Heavy metal pollution causes elevated concentrations of heavy metals in crops (44). As and Ni levels in vegetables are higher than normal values, and the estimated mean daily intakes of As and Pb in vegetables are above the permissible limits (45). In addition, corn kernels of Zn, Pb, Cd, Cr, and Ni exceeded the limits of China’s food hygiene standards (30). Thus, ducks live in the mining area with prolonged metal exposure inducing contaminated duck eggs. Particularly, differences in metal concentrations in duck egg yolks and whites are observed. Differences in the metal levels in duck egg yolks and whites might be related to their formation mechanisms. Once the female ducks absorb higher heavy metal levels they subsequently transfer to the embryo. Duck eggs are formed in the reproductive system of the duck and minerals are deposited into the eggs by two pathways, including ovary to yolk and oviduct to egg white (46, 47). The yolk precursor molecule of egg yolk protein could transfer minerals to the yolk, and the yolk component is synthesized in the liver, which is the main organ enrich heavy metals in the body (39, 40, 48). Therefore, most heavy metal levels are higher in egg yolks than in egg whites.

**Figure 4:**
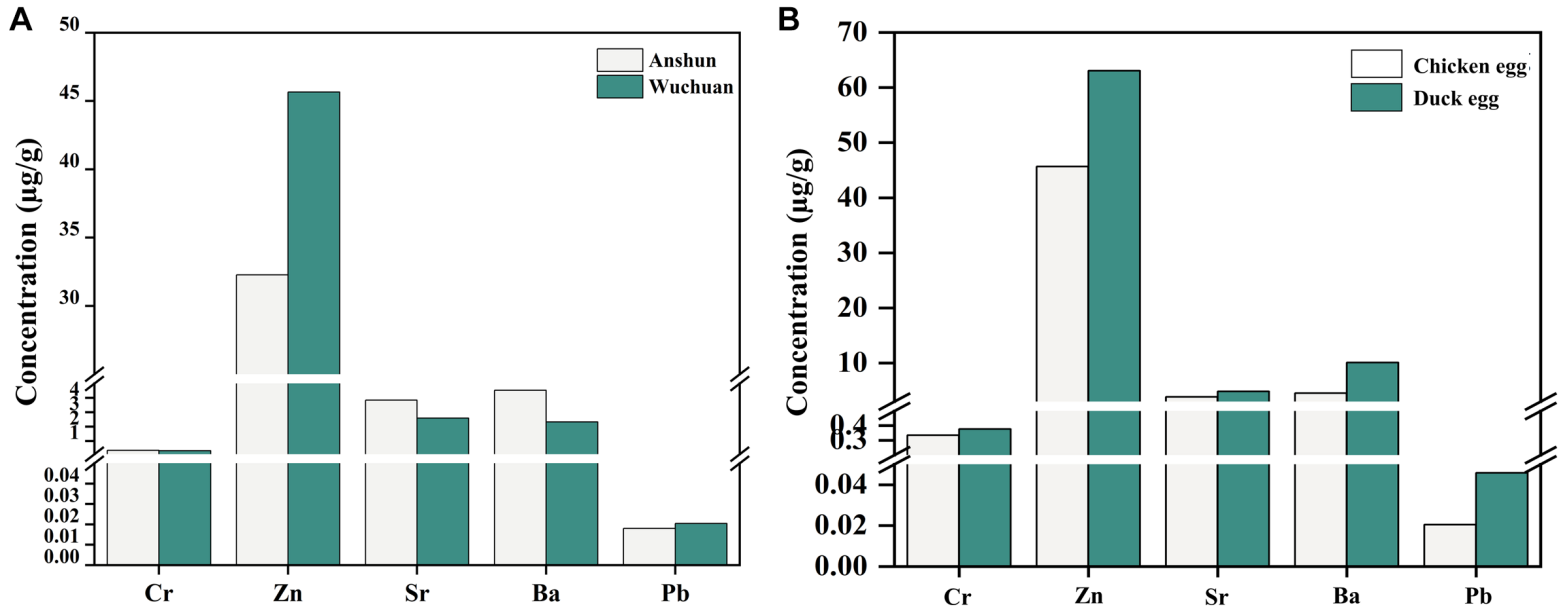
Heavy metal concentrations in duck eggs from mercury mining areas versus duck eggs from background areas **(A)**, and metal concentrations in eggs from mercury mining areas and duck eggs **(B)**.

We also measured Cr, Zn, Sr, Ba, and Pb concentrations in chicken eggs from the Wuchuan Hg mine, and the concentrations of Cr, Zn, Sr, Ba, and Pb in chicken eggs from the mine are not statistically different from those of the background area (*p* > 0.05; Figure 4A). Meanwhile, Cr, Zn, Sr, Ba, and Pb concentrations in duck eggs from the mining area are slightly higher than those in local chicken eggs (Figure 4B). Ducks belong to the waterfowl category of poultry, which eat not only crops, grasses earthworms but also fish and shrimps (49). Therefore, ducks are exposed to heavy metals from multiple sources with a possible higher heavy metal level compared to chickens and once ingested, heavy metals are enriched in the embryo then transferred to duck eggs, which may explain the higher heavy metal levels in duck eggs compared to chicken eggs (41, 49). Concentrations of Cr, Zn, Sr, Ba, and Pb in chicken eggs from the mine region do not differ from those in the background, indicating that chicken eggs are less contaminated than duck eggs in Hg mining region. However, the concentrations of Cr, Zn, Sr, Ba, and Pb in duck eggs in Hg mining area are higher than those in background area, suggesting that we should be more concerned about the potential risk of heavy metal contamination in duck eggs.

In addition, the sampling was performed in July, the rainy season in Guizhou. The heavy metals exposure to ducks are different between the dry and rainy seasons (50). Heavy metals accumulate in the environment during the dry season as a result of evaporation. Conversely, during the rainy season, the heavy metal exposure could be decreased due to dilution effects (51). Thus, high heavy metal levels in duck eggs during the rainy season in this study suggest even higher levels during the dry season. Notably, there is an ecological risk of heavy metal exposure in duck eggs. The duck eggs have been heavily contaminated with Ba, Pb, and Zn, with extremely strong potential ecological risk. Consistent with previous results on single factor pollution indices for crops in mining areas, the rice collected in the vicinity of the mining area is more severely contaminated by As, Sb, Cd, Cu, and Zn (52). Potatoes are heavily contaminated by heavy metals while cabbage and radish are lightly polluted (53, 54). The single factor of Pb, Cd, Cr, and Ni in maize seeds are all greater than 1, suggesting that all heavy metal contamination in the edible part of the crops has reached heavy contamination levels (52, 54). This result illuminates that mining area duck eggs, like crops, are ecologically risky.

### 4.2 Health risk of heavy metals to local residents

**Figure 5:**
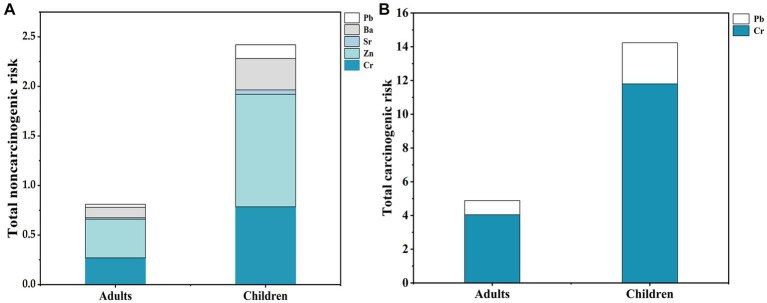
Total noncarcinogenic risk and total carcinogenic risk of heavy metals in adults and children. **(A)** Total noncarcinogenic risk of heavy metals in adults and children; **(B)** Total carcinogenic risk of heavy metals in adults and children.

The noncarcinogenic risk results suggest that non-age-specific, the total health risk index of egg yolk intake is >1. The contribution of the five heavy metals to the noncarcinogenic risk is illustrated in Figure 5A, Cr and Zn are the main noncarcinogenic risk metals for the inhabitants in the area, more significantly in children. The results indicate that noncarcinogenic health risks are associated with the consumption of duck eggs by both adults and children, and higher in children than in adults. Therefore, the noncarcinogenic risk of consuming duck eggs from Hg mining areas should not be ignored. Our results are consistent with crops in Hg mining areas, which indicate a health risk (55). Cr and Ni health risks are highest in maize from mining areas, and children are most sensitive to maize heavy metal exposure (55). The higher health risk of duck egg consumption in children than in adults suggests that children are more sensitive to environmental pollutions. Liver is the main organ that enriches and metabolism heavy metals (43, 53). However, children’s metabolic organs, such as the liver and kidney, are not yet well developed and have weaker detoxification functions for toxic and harmful substances (56). Whereas the health risks of egg yolks are greater than egg whites may be since the yolk protein precursor molecule in egg yolk can transfer minerals to the yolk, and the yolk component is synthesized in the liver (57). Therefore, concerns should be raised about the potential noncarcinogenic health risks to children from the consumption of mining area duck eggs, especially the yolks.

We find that the TCRs of Cr and Pb in duck eggs are greater than 1.00 × 10^−6^ when consumed by adults and children, indicating both have a certain carcinogenic risk from the intake of egg yolk and white. CR combined with TCR shows that Cr is the main contributing factor, indicating that Cr is the most significant carcinogenic risk metal in Hg mining area (Figure 5B). Long-term consumption of brown rice poses potential noncarcinogenic and carcinogenic health risks to the local population (43, 58). The same goes for long-term consumption of duck eggs. Although the Hg mining area is dominated by Hg pollution, the carcinogenic risk of Cr in local duck eggs should not be ignored. To sum up, duck eggs from Hg mining areas are contaminated with heavy metals and may pose a potential health risk to local residents who consume them.

Previous studies have reported high levels of Hg in the hair, blood, and urine of people living near the Wuchuan Hg mines (59, 60). It is suggested to be related to Hg pollution in this region. Except for Hg, high levels of other heavy metals have been observed in the mining areas, such as soil and vegetables (41, 44). According to our results in this study, high heavy metal concentrations in duck eggs indicate high levels of heavy metals in the environment and crops and further illustrate that local residents could possibly be exposed to high levels of heavy metals via poultry products (e.g., eggs) and environmental materials. Thus, the risk of heavy metal pollution posing to the residents is non-negligible.

### 4.3 Analysis of heavy metal concentrations in free-range and caged eggs

**Figure 6:**
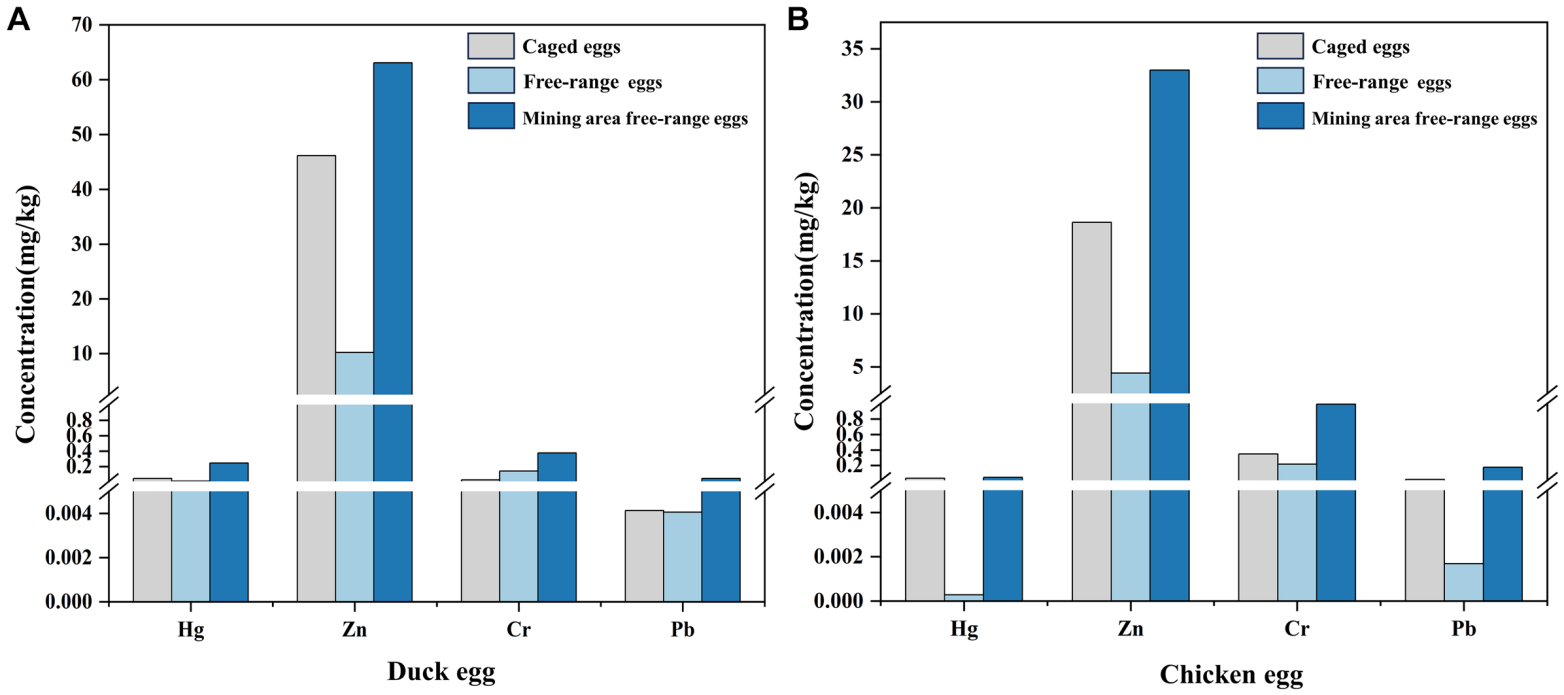
The concentrations of Hg, Zn, Cr, and Pb in free-range eggs and caged eggs (16, 17, 64–77). **(A)** Duck egg; **(B)** Chicken egg.

Eggs as the paramount source of protein consumption for humans, which could be roughly categorized into free-range eggs and caged eggs (61). Investigations have revealed a gradual increase in the overall consumption of eggs, with a growing preference for free-range eggs among consumers (62). During the same period, sales of free-range eggs in the Australian egg industry surge by 21.7%, while caged eggs show a decline of 12.5% (61). Similar preferences for free-range eggs have also been observed among consumers in various countries, including Canada and China, who perceive them to possess higher nutritional value and safety (10, 63). Based on comprehensive avian egg research (Figure 6), we categorized poultry eggs (duck egg and chicken egg) into caged eggs, background free-range eggs, and mining area free-range eggs. Interestingly, for duck eggs and chicken eggs, the concentrations of Cr, Zn, Sr, Ba, and Pb in background free-range eggs are found to be lower than those in caged eggs, which also elucidates why consumers favor free-range eggs. However, for free-range eggs from mining areas, the concentrations of Cr, Zn, Sr, Ba, and Pb are notably higher than those in caged eggs and background free-range eggs. In addition, consistent with previous studies, the heavy metal concentration in mining area of duck eggs is higher than chicken eggs (64, 65).

Disparities in heavy metal concentration among distinct egg types could be attributed to poultry rearing practices. Caged poultry are commonly provided with formulated feed, restricting their environmental exposure (78). On the other hand, free-range poultry predominantly feed on substances present in their surroundings, including insects and grains (63). Free-range poultry in mining areas feed on substances from their surrounding environment. It is widely recognized that mining areas face severe heavy metal pollution, with long half-lives and prolonged presence in the environment. Through the food chain, free-range poultry in mining areas are exposed to environmental heavy metal contamination, accumulating in their eggs. Therefore, the ingestion of mining area free-range eggs can pose a potential threat to human health. When choosing free range eggs, consumers should identify the producing areas.

## 5 Conclusion

In this study, we measured concentrations of five metals (Cr, Zn, Sr, Ba, and Pb) in duck eggs and chicken eggs from the Hg mining area and the background area, and found that duck eggs from the Hg mining area contained higher concentrations than those from the background area. Duck egg yolks contain higher concentrations than whites, which is related to the presence of yolk precursor proteins in the liver which is the main organ enrich heavy metals in the body. There is no difference in those metal concentrations between chicken eggs from Hg mining areas and background areas, which indicates that duck eggs are more susceptible to heavy metal contamination than chicken eggs. Duck eggs are contaminated by heavy metals to varying degrees, especially for Ba, Pb, and Zn, which have an extremely strong potential ecological risk. In view of different types of eggs from different areas, the concentration in free-range duck eggs and chicken eggs from mining areas are higher than that in farm and free-range duck eggs and chicken eggs. Therefore, when choosing free-range duck eggs as daily food, attention should be paid to identifying the producing regions, with a knowledge about the health risks of duck eggs from heavy metal contaminated areas, such as mining regions. Nevertheless, this is a preliminary study with limited number of duck egg samples. Further studies with increasing numbers of eggs and environmental (soil, water) and crop samples need to be performed to gain a better understanding of the sources of heavy metal pollution in duck eggs from Hg mining areas.

## Data availability statement

The original contributions presented in the study are included in the article/supplementary material, further inquiries can be directed to the corresponding author.

## Author contributions

XG: Conceptualization, Data curation, Formal analysis, Investigation, Methodology, Software, Writing – original draft, Writing – review & editing. ZhuW: Conceptualization, Formal analysis, Investigation, Supervision, Writing – review & editing. XL: Data curation, Investigation, Visualization, Writing – review & editing. JL: Investigation, Writing – review & editing. XZ: Methodology, Visualization, Writing – review & editing. YR: Investigation, Writing – review & editing. QW: Formal analysis, Methodology, Software, Writing – review & editing. TZ: Software, Visualization, Writing – review & editing. ZhoW: Methodology, Software, Writing – review & editing.
