# Supplementary material for: Heavy metal contamination in duck eggs from a mercury mining area, southwestern China
Source: Front Public Health. 2024 Feb 28;12:1352043. doi: 10.3389/fpubh.2024.1352043 (PMC10932963; doi:10.3389/fpubh.2024.1352043)
Supplement: Supplementary file 1 [file Table_1.DOCX]

Supplementary Material

# Supplementary Tables

## Supplementary Table S1

**Table S1.** Classification of heavy metal indexes

| **The range value of P_i_** | **Pollution levels classification** |
| --- | --- |
| *P_i_*≤1 | None |
| 1<*P_i_*≤2 | Slight pollution |
| 2<*P_i_*≤3 | Mild pollution |
| 3< *P_i_*≤5 | Moderate pollution |
| *P_i_*> 5 | Heavy pollution |

Note: *P_i_* the single-factor pollution index.
